# Urine creatinine concentration influences the prognostic value of proteinuria for MACE prediction from the findings of the KNOW-CKD study

**DOI:** 10.1038/s41598-022-19819-9

**Published:** 2022-09-23

**Authors:** Yun Jung Oh, Han Ro, Wookyung Chung, Young Youl Hyun, Sue Kyung Park, Yong-Soo Kim, Soo Wan Kim, Yun Kyu Oh, Kook-Hwan Oh, Ji Yong Jung

**Affiliations:** 1 Department of Internal Medicine, Graduate School of Medicine, Gachon University, Incheon, Republic of Korea; 2 Division of Nephrology, Department of Internal Medicine, H Plus Yangji Hospital, Seoul, Republic of Korea; 3 Division of Nephrology, Department of Internal Medicine, Gachon University Gil Medical Center, Incheon, Republic of Korea; 4 Gachon University College of Medicine, Incheon, Republic of Korea; 5 Department of Internal Medicine, Sungkyunkwan University School of Medicine, Kangbuk Samsung Hospital, Seoul, Republic of Korea; 6 Department of Preventive Medicine, Seoul National University College of Medicine, Seoul, Republic of Korea; 7 Department of Internal Medicine, Seoul St. Mary’s Hospital, Seoul, Republic of Korea; 8 Department of Internal Medicine, Chonnam National University Medical School, Gwangju, Republic of Korea; 9 Department of Internal Medicine, Seoul National University Boramae Hospital, Seoul, Republic of Korea; 10 Department of Internal Medicine, Seoul National University Hospital, Seoul, Republic of Korea; 11 Division of Nephrology, Department of Internal Medicine, Gachon University Gil Medical Center, Gachon University College of Medicine, 21, Namdong-daero 774 beon-gil, Namdong-gu, Incheon, 21565 Republic of Korea

**Keywords:** Medical research, Nephrology

## Abstract

Proteinuria is typically quantified according to the spot urine protein–creatinine ratio (UPCR) and an association with cardiovascular events has not been thoroughly investigated in chronic kidney disease (CKD) patients. We investigated whether the severity of proteinuria assessed by spot UPCR is associated with an increased risk for cardiovascular outcomes in the CKD population, and whether the relationship is influenced by urine creatinine concentration. We analyzed 1746 patients enrolled as part of The KoreaN cohort study for Outcome in patients With Chronic Kidney Disease (KNOW-CKD). Multivariable Cox proportional hazard analysis was performed to evaluate models with proteinuria as a predictor of renal events and extended major adverse cardiovascular events (eMACEs). Risk for renal events was significantly associated with proteinuria across all eGFR and UPCR categories. By contrast, risk for eMACEs increased significantly with UPCR in patients with eGFR ≥ 60 mL/min/1.73 m^2^ (hazard ratio [HR] 2.109; 95% confidence interval [CI] 1.375–3.235; P = 0.001), but not in patients with eGFR < 60 mL/min/1.73 m^2^ (HR 1.086; 95% CI 0.910–1.296; P = 0.358). However, in those with the lower eGFR, risk for eMACEs increased significantly with UPCR in participants with urine creatinine concentration ≥ 95 mg/dL (HR 1.503; 95% CI 1.047–2.159; P = 0.027). In non-dialysis CKD patients, the prognostic value of UPCR for eMACEs is weakened in patients with reduced eGFR levels, for whom it has prognostic significance only in patients with high urine creatinine concentration.

## Introduction

Proteinuria is a risk factor for the progression of chronic kidney disease (CKD) and is associated with an increase in cardiovascular complications^1–5^. An association between increased proteinuria and risk for cardiovascular complications has been found in both the general population^2,3,6^ and subgroup populations with established risk factors such as diabetes^7^ and hypertension^8^. However, whether the risk for cardiovascular events (CVEs) increases in proportion to the severity of proteinuria in CKD patients has not been investigated thoroughly. In a collaborative meta-analysis of the general population, cardiovascular mortality increased with the category of albuminuria, but this association tended to be weak in individuals with estimated glomerular filtration rate (eGFR) < 45 mL/min/1.73 m^2^, with a wide 95% confidence interval (CI) overlapping albuminuria categories^2^. In addition, another collaborative meta-analysis of high-risk group cohorts including patients with hypertension, diabetes, or cardiovascular disease similarly reported that higher albuminuria was associated with increased cardiovascular mortality, but the association tended to be weak in patients with severely reduced eGFR^4^.

To quantify proteinuria, the gold standard method is to measure the 24 h urinary protein excretion (UPE). However, collecting urine output for 24 h is inconvenient and the results are often inaccurate due to errors in collection. Thus, in current clinical practice, the spot urine protein–creatinine ratio (UPCR) is mainly used for proteinuria quantification based on the finding that spot UPCR correlates well with 24 h UPE^9,10^. In calculating the spot UPCR, urine creatinine concentration is used in the denominator to correct for the effects of urine tonicity. However, urine creatinine is affected by muscle mass as well as urine tonicity, and muscle mass is affected by sex, age, nutritional status, and comorbid medical conditions^11–13^. In other words, urine creatinine concentration may have indirect prognostic information on cardiovascular outcomes and influence the association between UPCR and these outcomes. Previous studies showing that lower urine creatinine excretion is associated with a higher risk for CVEs and mortality^14–16^ support this assumption. However, the potential influence of urine creatinine concentration on the association between UPCR and cardiovascular outcomes has not yet been investigated in CKD patients.

In this study, we investigated whether the severity of proteinuria assessed by spot UPCR is associated with increased risk for cardiovascular outcomes, and whether the relationship is influenced by urine creatinine concentration in the CKD population.

## Results

### Baseline characteristics of the study population

A total of 1746 participants with CKD were included in the study; the whole cohort has a mean eGFR of 53.4 ± 30.9 mL/min/1.73 m^2^, a mean age of 53.9 ± 12.1 years, and 60.0% of the participants were male. The median spot UPCR was 500 mg/g (interquartile range, 100–1400 mg/g), 11.6% of the participants had severely increased proteinuria (UPCR ≥ 3000 mg/g) and 38.3% had mild proteinuria (UPCR < 300 mg/g). Overall, 10.3% of all of the participants had diabetes and 23.4% had glomerulonephritis; 85.7% of all of the participants were prescribed renin–angiotensin–aldosterone system blocker at enrollment. The baseline characteristics of the participants grouped by UPCR category are listed in Table 1. Participants with higher proteinuria were older and more likely to have diabetes, hypertension, and lower eGFR levels, compared to those with lower proteinuria.

**Table 1:** Baseline characteristics of study participants.

UPCR (mg/g)						
	< 300 (n = 669)	300–999 (n = 497)	1000–2999 (n = 377)	≥ 3000 (n = 203)	Total (n = 1746)	*P*
Age, year	53.2 ± 12.2	53.7 ± 12.1	54.5 ± 12.1	55.4 ± 11.2	53.9 ± 12.1	0.012
Male gender, n (%)	415 (62.0%)	289 (58.1%)	212 (56.2%)	131 (64.5%)	1047 (60.0%)	0.120
BMI, kg/m^2^	24.3 ± 3.3	24.6 ± 3.2	24.8 ± 3.6	25.4 ± 3.5	24.6 ± .3.4	< 0.001
Smoking, n (%)	314 (46.9%)	226 (45.5%)	176 (46.7%)	100 (49.3%)	816 (46.7%)	0.838
Diabetes, n (%)	149 (22.3%)	151 (30.4%)	158 (41.9%)	150 (73.9%)	608 (34.8%)	< 0.001
Hypertension, n (%)	616 (92.1%)	485 (97.6%)	370 (98.1%)	201 (99.0%)	1672 (95.8%)	< 0.001
Previous CVD, n (%)	61 (9.1%)	49 (9.9%)	43 (11.4%)	26 (12.8%)	179 (10.3%)	0.391
Glomerulonephritis, n (%)	99 (14.8%)	158 (31.8%)	118 (31.3%)	34 (16.7%)	409 (23.4%)	< 0.001
eGFR, ml/min/1.73 m^2^	65.1 ± 30.1	52.7 ± 30.1	43.8 ± 28.9	34.4 ± 22.8	53.4 ± 30.9	< 0.001
Hemoglobin, g/dl	13.4 ± 1.8	12.9 ± 2.0	12.4 ± 2.0	11.5 ± 1.8	12.8 ± 2.0	< 0.001
Albumin, g/dl	4.4 ± 0.3	4.3 ± 0.3	4.1 ± 0.3	3.6 ± 0.5	4.2 ± 0.4	< 0.001
Cholesterol, mg/dl	173.3 ± 34.9	167.5 ± 34.6	175.5 ± 40.4	186.2 ± 50.7	173.6 ± 38.5	< 0.001
hs-CRP, mg/dl	2.0 ± 5.6	2.1 ± 5.4	2.0 ± 4.6	1.8 ± 3.4	2.0 ± 5.1	0.817
Calcium, mg/dl	9.3 ± 0.4	9.2 ± 0.5	9.0 ± 0.5	8.6 ± 0.7	9.1 ± 0.5	< 0.001
Phosphorus, mg/dl	3.5 ± 0.6	3.7 ± 0.6	3.8 ± 0.7	4.1 ± 0.8	3.7 ± 0.7	< 0.001
RAS bloker, n (%)	545 (81.5%)	443 (89.1%)	324 (85.8%)	185 (01.1%)	1497 (85.7%)	< 0.001
Statin, n (%)	266 (21.4%)	285 (57.3%)	220 (58.4%)	142 (70.0%)	913 (52.3%)	< 0.001
UPCR (mg/g)	100 (100–200)	500 (400–700)	1700 (1300–2200)	5000 (3700–7000)	500 (100–1400)	< 0.001
UPE (mg/day)	100 (100–300)	600 (400–900)	1700 (1100–2400)	4700 (3200–7200)	600 (200–1600)	< 0.001

*UPCR* urine protein-creatinine ratio, *BMI* body mass index, *CVD* cardiovascular disease, *RAS* renin–angiotensin–aldosterone system; UPE, urine protein excretion.

### Associations between proteinuria and eGFR levels and risk for eMACEs and renal events in CKD patients

During follow up, with a median time of 73.1 months, 142 eMACEs and 604 renal events occurred. Table 2 lists the unadjusted and adjusted risks of eMACEs and renal events for the 20 categories of eGFR and proteinuria. Using the category with eGFR ≥ 90 mL/min/1.73 m^2^ and UPCR < 300 mg/g as the reference, the unadjusted HR for eMACEs increased with proteinuria level and decreased with eGFR level in patients with an eGFR of 15–90 mL/min/1.73 m^2^. However, the adjusted risk for eMACEs was significantly increased in patients in the highest proteinuria category (UPCR ≥ 3000 mg/g) and eGFR ≥ 60 mL/min/1.73 m^2^. By contrast, the risk for renal events was significantly associated with elevated proteinuria across all eGFR and UPCR categories in both unadjusted and adjusted Cox regression analyses.

**Table 2:** Hazard ratios (with 95% confidence interval) for extended major adverse cardiovascular events (eMACEs) and renal events according to category of estimated glomerular filtration rate (eGFR) and urine protein-creatinine ratio (UPCR).

	Unadjusted HR (95% CI)				Adjusted HR* (95% CI)			
	UPCR (mg/g)				UPCR (mg/g)			
	< 300	300–999	1000–2999	≥ 3000	< 300	300–999	1000–2999	≥ 3000
**eMACE**								
≥ 90 mL/min/1.73 m^2^	Reference	0.69 (0.14–3.43)	0.88 (0.11–7.30)	3.28 (0.39–27.26)	Reference	1.45 (0.25–8.45)	0.84 (0.07–9.77)	38.89 (1.63–976.19)
60–89 mL/min/1.73 m^2^	2.34 (0.90–6.09)	1.17 (0.62–2.20)	1.83 (1.01–3.23)	2.70 (1.43–5.08)	1.32 (0.46–3.79)	1.13 (0.56–2.28)	1.67 (0.82–3.42)	4.74 (1.14–19.65)
30–59 mL/min/1.73 m^2^	1.45 (0.91–2.30)	1.54 (1.14–2.07)	1.18 (0.83–1.68)	1.63 (1.15–2.30)	0.88 (0.49–1.56)	1.32 (0.92–1.89)	0.85 (0.53–1.38)	1.39 (0.79–2.44)
15–29 mL/min/1.73 m^2^	1.67 (1.19–2.34)	1.23 (0.94–1.60)	1.38 (1.08–1.76)	1.37 (1.04–1.80)	1.32 (0.82–2.12)	0.95 (0.67–1.33)	1.13 (0.81–1.59)	1.04 (0.69–1.57)
< 15 mL/min/1.73 m^2^	1.24 (0.73–2.10)	1.05 (0.69–1.61)	1.11 (0.81–1.53)	1.27 (0.96–1.68)	1.06 (0.57–1.99)	0.96 (0.59–1.56)	0.94 (0.61–1.45)	1.06 (0.69–1.62)
**Renal event**								
≥ 90 mL/min/1.73 m^2^	Reference	4.56 (1.31–15.84)	2.97 (0.54–16.30)	27.46 (6.14–122.90)	Reference	9.38 (1.62–54.22)	1.09 (0.15–8.03)	69.00 (14.60–326.21)
60–89 mL/min/1.73 m^2^	4.27 (1.41–12.92)	2.89 (1.62–5.14)	4,28 (2.41–7.60)	4.99 (2.49–9.97)	7.01 (1.93–25.53)	4.72 (2.10–10.58)	5.41 (2.67–10.96)	5.51 (2.72–11.13)
30–59 mL/min/1.73 m^2^	2.69 (1.60–4.52)	2.72 (1.93–3.82)	3.38 (2.41–4.76)	4.25 (2.86–6.29)	4.40 (2.47–7.84)	3.05 (2.09–4.46)	3.47 (2.40–5.00)	4.35 (2.75–6.87)
15–29 mL/min/1.73 m^2^	3.33 (2.24–4.95)	2.55 (1.98–3.29)	3.29 (2.45–4.44)	3.49 (2.68–4.56)	4.71 (2.89–7.67)	2.69 (2.04–3.56)	3.30 (2.37–4.61)	3.82 (2.73–5.35)
< 15 mL/min/1.73 m^2^	15.67 (0.01–24,630)	3.47 (2.31–5.21)	2.73 (2.14–3.49)	3.55 (2.36–5.34)	6.30 (2.06–19.26)	4.70 (2.64–8.39)	3.19 (2.48–4.10)	6.11 (3.09–12.10)

*HR* hazards ratio, *CI* confidence interval, *UPCR* urine protein-creatinine ratio, *eMACE* extended major adverse cardiovascular events.

*Adjusted for age, sex, BMI, smoking, previous CVD, diabetes, hypertension, glomerulonephritis, and use of RAS blocker and statin.

### Risk for eMACEs and renal events according to severity of proteinuria quantified by UPCR in CKD patients

We evaluated whether the risk for eMACEs increased proportionally with the degree of proteinuria, as quantified by UPCR using Cox regression models. UPCR revealed a highly skewed distribution, therefore we calculated HRs using 1-standard deviation (SD) increase unit for UPCR in regression analyses. Table 3 lists the adjusted HR for eMACEs per 1-SD unit increase in UPCR. Overall, the risk for eMACEs seemed to increase with UPCR, but the trend did not reach statistical significance (HR 1.143; 95% CI 0.979–1.334, P = 0.091). However, when evaluated within subgroups defined relative to eGFR = 60 mL/min/1.73 m^2^, the risk for eMACEs increased significantly with UPCR in patients with eGFR ≥ 60 mL/min/1.73 m^2^ (HR 2.109; 95% CI 1.375–3.235; P = 0.001), but this was not significantly associated with UPCR in those with eGFR < 60 mL/min/1.73 m^2^ (HR 1.086; 95% CI 0.910–1.296; P = 0.358). To evaluate an effect modification of the association between UPCR and risk for eMACEs due to the difference of eGFR levels (being above or below eGFR = 60 mL/min/1.73 m^2^), we conducted a test for interaction using multivariable models. The significant P value for interaction of subgroups by being above or below eGFR = 60 mL/min/1.73 m^2^ (P = 0.011) indicates that the predictive effect of UPCR for eMACEs differs depending on the eGFR levels. Additionally, we evaluated the predictive value of UPCR for eMACEs in further subdivided three groups (eGFR ≥ 60 mL/min/1.73 m^2^, eGFR 45–59 mL/min/1.73 m^2^, and eGFR < 45 mL/min/1.73 m^2^). The predictive value of UPCR for eMACEs was significant only in eGFR ≥ 60 mL/min/1.73 m^2^ (Table S1) as well. The risk for renal events significantly increased with UPCR in both overall (HR 1.809; 95% CI 1.697–1.929; P < 0.001) and in subgroup analyses (HR 1.542; 95% CI 1.170–2.032; P = 0.002 with eGFR ≥ 60 mL/min/1.73 m^2^ and HR 1.838; 95% CI 1.717–1.968; P < 0.001 with eGFR < 60 mL/min/1.73 m^2^).

**Table 3:** Adjusted hazard ratios of per SD-unit increase in urine protein-creatinine ratio (UPCR) for extended major adverse cardiovascular events (eMACEs) and renal events.

Overall (n = 1746)	HR (95% CI)	*P*	
eMACE	1.143 (0.979–1.334)	0.091	
Renal event	1.809 (1.697–1.929)	< 0.001	

eMACE	HR (95% CI)	*P*	
		Effect	Interaction
eGFR ≥ 60 mL/min/1.73 m^2^ (n = 618)	2.109 (1.375–3.235)	0.001	0.011
eGFR < 60 mL/min/1.73 m^2^ (n = 1128)	1.086 (0.910–1.296)	0.358	

Renal event	HR (95% CI)	*P*	
		Effect	Interaction
eGFR ≥ 60 mL/min/1.73 m^2^ (n = 618)	1.542 (1.170–2.032)	0.002	0.088
eGFR < 60 mL/min/1.73 m^2^ (n = 1128)	1.838 (1.717–1.968)	< 0.001	

*HR* hazards ratio, *CI* confidence interval, *UPCR* urine protein-creatinine ratio, *eMACE* extended major adverse cardiovascular events.

*Adjusted for age, sex, BMI, smoking, previous CVD, diabetes, hypertension, glomerulonephritis, baseline eGFR, and use of RAS blocker and statin.

### Associations between UPCR and risk for eMACEs and renal events stratified by urine creatinine concentration

**Figure 1:**
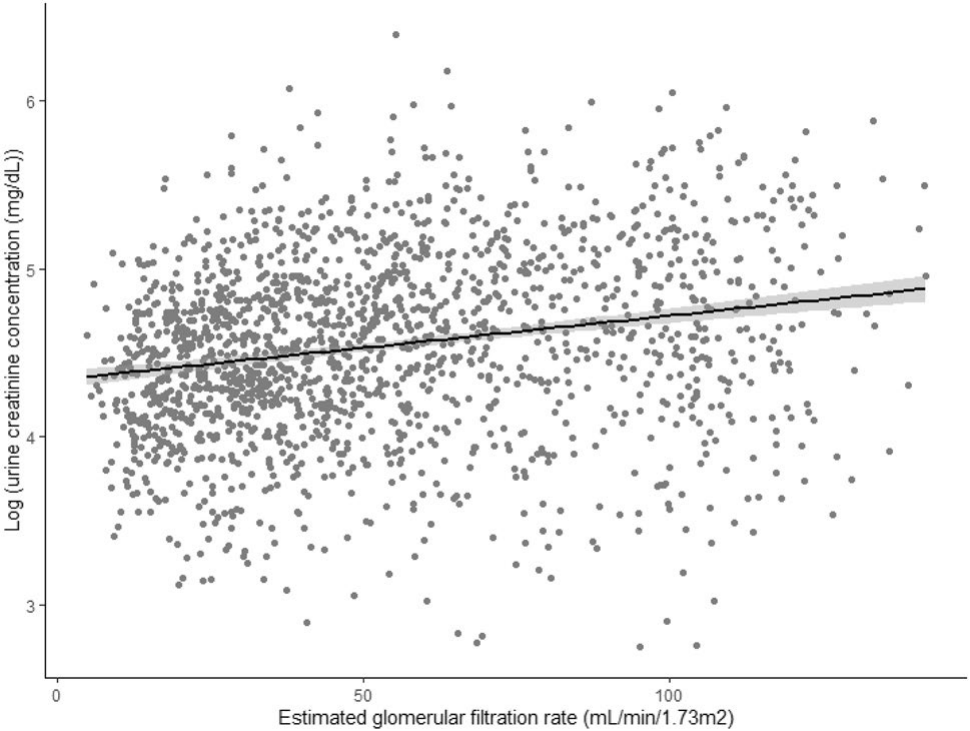
Scatter plot of estimated glomerular filtration rate (eGFR) versus urine creatinine concentration on a log scale. The linear regression is shown as a solid line.

**Figure 2:**
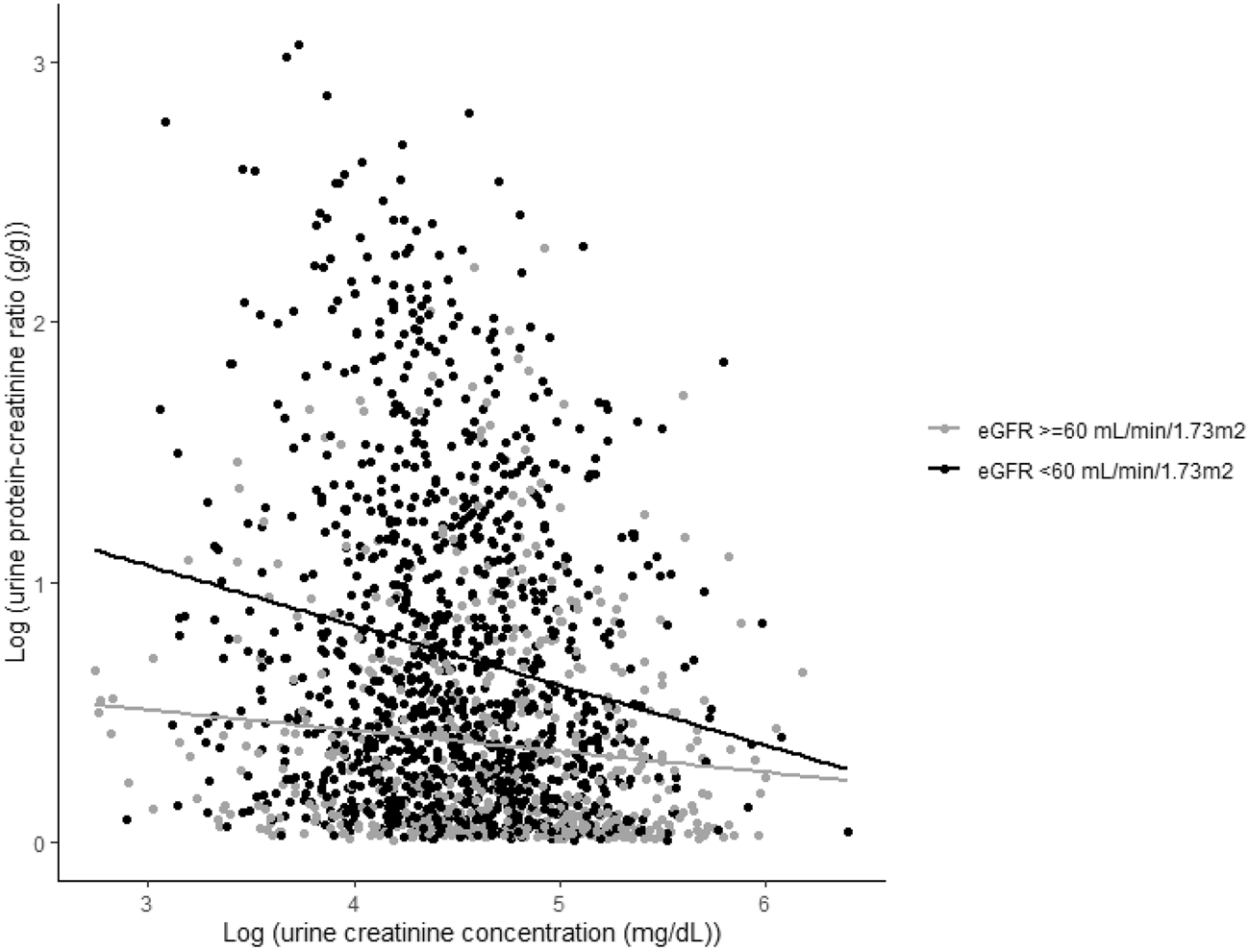
Scatter plot of urine creatinine concentration versus urine protein–creatinine ratio (UPCR; on log–log scales) stratified by estimated glomerular filtration rate (eGFR) category. This shows an inverse correlation between urine creatinine concentration and UPCR in patients with eGFR ≥ 60 or < 60 mL/min/1.73 m^2^. The linear regression is shown as a gray (black) solid line for the former (latter).

**Figure 3:**
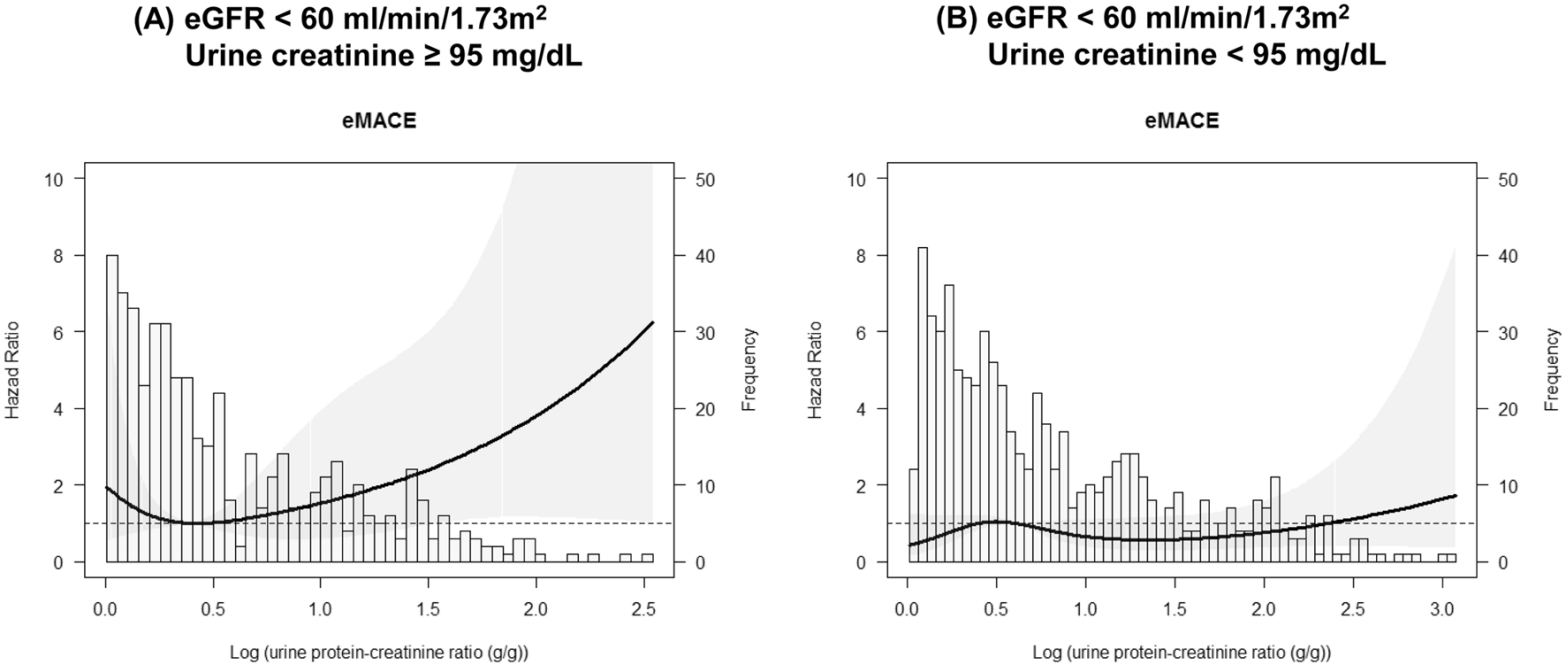
Adjusted risk for extended major adverse cardiovascular events (eMACEs) according to urine protein–creatinine ratio (UPCR) on a log scale in patients with estimated glomerular filtration rate (eGFR) < 60 mL/min/1.73 m^2^ stratified by urine creatinine concentration. Spline curves showing the adjusted hazard ratios of eMACEs in patients with urine creatinine concentration ≥ 95 (**A**) and < 95 mg/dL (**B**). These ratios were adjusted for age; sex; body mass index; smoking status; presence of diabetes, hypertension, glomerulonephritis, and previous cardiovascular disease; baseline eGFR; and use of renin–angiotensin–aldosterone system blocker and statins. The histograms show the frequency of distribution of spot UPCR.

Figure 1 shows a scatter plot of eGFR versus log-transformed urine creatinine concentration; they correlate positively. Figure 2 shows a scatter plot of urine creatinine concentration versus UPCR on log scales, stratified by eGFR category. The inverse relationship between urine creatinine concentration and UPCR is expected because the former is the denominator in the formula of the latter. However, the strength of this relationship differed somewhat between the two groups categorized as being above or below eGFR = 60 mL/min/1.73 m^2^ with the association being much stronger in individuals with lower eGFR. To evaluate the influence of urine creatinine concentration on the relationship between UPCR and eMACEs, we analyzed this association in groups stratified by urine creatinine concentration. The median urine creatinine concentration 94.0 mg/dL; hence, we conducted Cox regression analyses in two groups categorized as being above or below urine creatinine concentration = 95 mg/dL. In participants with eGFR ≥ 60 mL/min/1.73 m^2^, the adjusted risk for eMACEs increased proportionally and significantly with UPCR in both groups (urine creatinine concentration ≥ 95 mg/dL, HR 2.364; 95% CI 1.337–4.183, P = 0.003; and < 95 mg/dL, HR 2.629; 95% CI 1.121–6.167, P = 0.026; Table 4). However, in those with eGFR < 60 mL/min/1.73 m^2^, the risk for eMACEs increased significantly with UPCR only in participants with urine creatinine concentration ≥ 95 mg/dL (HR 1.503; 95% CI 1.503–2.159; P = 0.027); the risk for eMACEs was not significantly associated with UPCR in participants with urine creatinine concentration < 95 mg/dL (HR 1.013; 95% CI 0.817–1.257; P = 0.906). P value for interaction of being above or below urine creatinine concentration = 95 mg/dL was significant (P = 0.026) supporting that the predictive value of UPCR for eMACEs differed within the subgroups with eGFR < 60 mL/min/1.73 m^2^. Figure 3 shows an adjusted HR curve for eMACEs according to log-transformed UPCR in two groups categorized by whether eGFR is above or below 60 mL/min/1.73 m^2^. The HR appears to increase according with UPCR in individuals with eGFR ≥ 60 mL/min/1.73 m^2^, while it seems to vary little those with eGFR < 60 mL/min/1.73 m^2^. The risk for renal events increased proportionally with UPCR regardless of urine creatinine concentration in both groups, with eGFR ≥ 60 mL/min/1.73 m^2^ and eGFR < 60 mL/min/1.73 m^2^.

**Table 4:** Adjusted hazard ratios of per SD-unit increase in urine protein-creatinine ratio (UPCR) for extended major adverse cardiovascular events (eMACEs) and renal events stratified by urine creatinine concentration across the whole study population.

eMACE				Renal event			
	HR (95% CI)	*P*			HR (95% CI)	*P*	
		Effect	Interaction			Effect	Interaction
**eGFR ≥ 60 mL/min/1.73 m**^**2**^** (n = 618)**							
Urine creatinine ≥ 95 mg/dL	2.364 (1.337–4.183)	0.003	0.993	Urine creatinine ≥ 95 mg/dL	1.612 (1.110–2.342)	0.012	0.659
Urine creatinine < 95 mg/dL	2.629 (1.121–6.167)	0.026		Urine creatinine < 95 mg/dL	2.117 (1.210–3.705)	0.009	
**eGFR < 60 mL/min/1.73 m**^**2**^** (n = 1128)**							
Urine creatinine ≥ 95 mg/dL	1.503 (1.047–2.159)	0.027	0.026	Urine creatinine ≥ 95 mg/dL	2.239 (1.909–2.625)	< 0.001	< 0.001
Urine creatinine < 95 mg/dL	1.013 (0.817–1.257)	0.906		Urine creatinine < 95 mg/dL	1.768 (1.631–1.916)	< 0.001	

*HR* hazards ratio, *CI* confidence interval, *UPCR* urine protein-creatinine ratio, *eMACE* extended major adverse cardiovascular event.

*Adjusted for age, sex, BMI, smoking, previous CVD, diabetes, hypertension, glomerulonephritis, baseline eGFR, and use of RAS blocker and statin.

### Sensitivity analysis

We evaluated the predictive value of spot urine albumin–creatinine ratio (UACR) for eMACEs and renal events and found that the results were similar to those of UPCR except that the association between UACR and adjusted HR for eMACEs was not significant in individuals with eGFR < 60 mL/min/1.73 m^2^ and urine creatinine concentration ≥ 95 mg/dL (Tables S2–S4). UACR was also positively and significantly associated with risk for eMACEs in individuals with eGFR ≥ 60 mL/min/1.73 m^2^ (HR 2.107; 95% CI 1.388–3.197, P = 0.001), but no significant relationship between UACR and risk for eMACEs was observed in individuals with eGFR < 60 mL/min/1.73 m^2^ (HR 1.069; 95% CI 0.895–1.277, P = 0.461). P for interaction of being above or below eGFR = 60 mL/min/1.73 m^2^ was significant (P = 0.010). However, UACR was a significant predictor of renal events irrespective of eGFR as with UPCR. These findings were also observed between the 24 h UPE level and eMACEs and renal events (data not shown).

## Discussion

In this prospective CKD cohort study, we found that higher spot UPCR was associated with increased risk for eMACEs in CKD patients with eGFR ≥ 60 mL/min/1.73 m^2^, whereas higher UPCR was not a predictor for risk for eMACEs in CKD patients with eGFR < 60 mL/min/1.73 m^2^. However, the predictive value of UPCR for this risk remained in the subgroup of CKD patients with eGFR < 60 mL/min/1.73 m^2^, who had high urine creatinine concentration (> 95 mg/dL). In terms of the relationship between UPCR and renal outcomes, higher UPCR was consistently associated with an increased risk for renal events regardless of eGFR and urine creatinine concentration levels.

Prior studies have demonstrated that higher levels of proteinuria are significantly associated with increased risks of mortality and CVEs, as well as kidney disease progression, suggesting that the severity of proteinuria is an important determinant of cardiovascular and renal outcomes^1–8^. However, considering that CKD patients are already likely to have proteinuria compared to the general population or other disease-related risk populations, it has not been conclusively determined whether the severity of proteinuria provides prognostic information for cardiovascular outcomes in CKD patients. In terms of the relationship between severity of proteinuria and renal outcomes, several studies conducted in CKD patients or populations with increased risk for CKD have reported that higher proteinuria is associated with increased risk for end-stage renal disease and CKD progression^3–5^. Our present findings, conducted in a CKD cohort, are consistent with these results, confirming that severity of proteinuria is a strong predictor for renal outcomes in CKD patients.

However, regarding the relationship between severity of proteinuria and cardiovascular outcomes, the findings observed in populations with reduced eGFR differ somewhat compared to those in the general population. A previous collaborative meta-analysis, despite being conducted for the general population, found that the association between increased risk for cardiovascular mortality and increased proteinuria seemed to be weaker or entirely unclear in participants with severely reduced eGFR^2^. Similarly, a previous study conducted on a population at high risk for CKD found that the relationship between proteinuria severity and cardiovascular mortality risk in individuals with markedly reduced eGFR also tended to be weaker^4^. Furthermore, a collaborative meta-analysis of CKD cohorts demonstrated that higher proteinuria was much more strongly associated with the risk for end-stage renal disease than with the risk for mortality in participants with CKD^5^. Likewise, our study shows that higher proteinuria is significantly associated with increased risk for renal outcomes across a wide range of eGFR in the CKD cohort, while higher proteinuria is associated with increased risk for CVEs only in the early-stage CKD patients, which indicates that severity of proteinuria is still a strong predictor of renal outcome in CKD patients as a whole, but we presume that its predictive power for CVEs is weaker in CKD patients with reduced eGFR. There were no significant differences in the associations of UPCR, UACR, or 24 h UPE with renal events or eMACEs, suggesting that these measurements all provide similar prognostic information in CKD patients. Proteinuria is an important sign of kidney damage with numerous clinical and experimental studies having proved that proteinuria plays a key role in kidney injury and its progression. Sustained proteinuria contributes to glomerular impairment and tubulointerstitial injury by multiple mechanisms, including change in the glomerular filtration barrier and induction of chemokine expression and complementary activation that can lead to tubulointerstitial inflammation and fibrosis^17–21^. In other words, proteinuria is a direct primary factor for renal injury and progression, which explains the strong association between higher proteinuria and increased risk for renal outcomes. By contrast, when it comes to CVEs, proteinuria is one of numerous comorbid conditions that influence cardiovascular outcomes. Although many studies have demonstrated that proteinuria is associated with not only several surrogate outcomes of cardiovascular disease but also cardiovascular mortality^2,4,6–8,22,23^ the exact mechanism by which proteinuria leads to CVEs is not clear. Compared to the direct relationship between proteinuria and renal outcome, proteinuria seems to be an indirect risk factor for CVEs. Particularly, CKD patients commonly have multiple comorbidities, besides proteinuria, that may affect cardiovascular outcomes, and it is highly probable that they will be exposed to more cardiovascular risk factors as CKD progresses. Therefore, we may assume that the influence of proteinuria on CVEs becomes weaker as kidney function declines. This may explain our finding that proteinuria could not predict the risk for CVEs in CKD patients with decreased eGFR.

The present study shows that the association between higher UPCR and greater risk for CVEs is maintained in CKD patients with eGFR < 60 mL/min/1.73 m^2^ who have urine creatinine concentration ≥ 95 mg/dL, but this association was not observed in those with eGFR < 60 mL/min/1.73 m^2^ and urine creatinine concentration < 95 mg/dL. This suggests that the association between UPCR and cardiovascular risk is modified by urine creatinine concentration in CKD patients with reduced eGFR. Similarly, previous studies have suggested that urine creatinine might influence the relationship between UACR and CVEs. The findings of previous studies conducted in community-living populations suggested that low urine creatinine concentration may be an important factor in the relationship between UACR and cardiovascular risk^24,25^. The authors hypothesized that spot UACR may be more strongly associated with risk for CVEs in participants with low muscle mass because low urine concentration may reflect low muscle mass and this is also associated with increased cardiovascular risk. Therefore, low urine creatinine concentration being in the denominator for calculating UACR may bias UACR to a higher level than it truly has, and the relationship between higher UACR and increased cardiovascular risk may be attributable not to the UACR level but instead to the low muscle mass associated with low urine creatinine concentration. However, against the authors’ expectations, their studies demonstrated that, although muscle mass is associated with urine creatinine concentration, the association between UACR and CVEs is driven primarily by urine albumin concentration rather than urine creatinine concentration. This finding is different from our present one that the association between UPCR and CVEs depends on urine creatinine concentration, which may arise from the different study populations. While previous studies were conducted in generally healthy, community-living populations with 24 h urine albumin excretion < 30 mg/day or without cardiovascular disease at baseline, our study was performed in CKD patients who mostly had proteinuria and several medical comorbidities at enrollment. The influence on the estimated value of UACR of urine creatinine concentration being in its denominator may be smaller in a generally healthy, community-living population with normoalbuminuria or microalbuminuria than in CKD patients who commonly have overt proteinuria, rather than microalbuminuria. When calculating UACR or UPCR, urine creatinine concentration is used in the denominator to correct the effect of urine tonicity. Urine concentration ability is decreased in patients with CKD and the severity of deterioration varies according to the etiology of CKD and degree of renal impairment^26–28^. Therefore, in CKD patients, particularly those with more severe renal impairment, the urine creatinine concentration may be more likely to influence the association between UPCR and CVEs. However, these speculations are merely hypothetical for the present findings; the underlying mechanisms are uncertain.

Although we conducted the present study using a large-scale prospective CKD cohort across all stages of the disease, our study had some limitations. It was conducted only in a Korean CKD cohort and thus our findings are limited in how they can be generalized to other racial and ethnic groups. In addition, our analyses did not reflect any individual’s change in UPCR during follow-up. Finally, we lacked other measurements to evaluate urine tonicity, such as urine specific gravity or osmolality, for comparison with urine creatinine concentration.

In conclusion, higher spot UPCR is associated with increased risk for eMACEs in CKD patients with eGFR ≥ 60 mL/min/1.73 m^2^, but the predictive power of this is attenuated in CKD patients with eGFR < 60 mL/min/1.73 m^2^, for whom higher UPCR provides significant prognostic information for eMACEs only in CKD patients with urine creatinine concentration ≥ 95 mg/dL. Therefore, these results suggest that attention should be paid to interpreting UPCR results as predictor of MACEs in CKD patients with reduced eGFR levels and that these should be considered together with urine creatinine concentrations.

## Methods

### Study design and participants

The KoreaN cohort study for Outcome in patients with Chronic Kidney Disease (KNOW-CKD) is an ongoing nationwide, multicenter, and prospective observational cohort study. Its detailed methods have previously been described^29^. In brief, 2238 adults aged between 20 and 75 years with CKD at stages G1–G5 (non-dialysis) due to various causes were enrolled between 2011 and 2016 (NCT01630486 at http://www.clinicaltrials.gov). Among them, 374 participants without baseline spot UPCR or 24 h UPE and 118 participants without measurements of other baseline covariates were excluded. Ultimately, 1746 participants were included in the final analysis. This study was conducted in accordance with the Declaration of Helsinki, and the research protocol was approved by the institutional review boards of the Seoul National University Hospital (1104-089-359), Seoul National University Bundang Hospital (B-1106/129–008), Yonsei University Severance Hospital (4-2011-0163), Kangbuk Samsung Medical Center (2011-01-076), Seoul St. Mary’s Hospital (KC11OIMI0441), Gachon University Gil Medical Center (GIRBA2553), Eulji General Hospital (201105-01), Chonnam National University Hospital (CNUH-2011-092), and Pusan Paik Hospital (11–091). Written informed consent was obtained from all subjects.

### Data collection and measurements

Demographic information including sex, age, smoking status, medication use, and medical history were obtained at enrollment by self-reported questionnaire. Anthropometric data such as body weight and height were collected at baseline, and body mass index was calculated using body weight divided by height (kg/m^2^). For serum creatinine measurement and proteinuria quantification, after overnight fasting, blood and urine samples were collected, and aliquots of the samples were sent to the central laboratory of KNOW-CKD. The other biochemical parameters were measured at each local participating institution. We used the Chronic Kidney Disease Epidemiology Collaboration creatinine equation^30^ to calculate eGFR. Participants were followed up regularly in accordance with study protocol, and the events data for study outcomes were recorded during these follow-ups. Information on mortality was investigated by reviewing medical records or data from the National Database of Statistics Korea. In the case of participants who were lost to follow up, information on survival and cause of death has been traced with the help of the National Health Insurance system of South Korea and Korea Statistical Information Service.

### Outcomes

The primary outcome was extended major adverse cardiovascular events (eMACEs), which was defined as cardiovascular events (fatal or non-fatal) including acute myocardial infarction; hospitalization for unstable angina or heart failure; percutaneous coronary intervention or coronary artery bypass graft; ischemic or hemorrhagic stroke; symptomatic arrhythmia that required hospitalization; peripheral arterial disease; and other CVEs that required hospitalization or interventional treatment. The secondary outcome was renal events, which included a decline of eGFR from baseline of > 50%; doubling of serum creatinine; and development of kidney failure that required renal replacement therapy.

### Statistical analyses

The data are described as mean ± standard deviation for continuous variables and frequencies and percentages for categorical variables. Initial analyses were performed to investigate the joint association between eGFR and proteinuria and outcomes such as eMACEs and renal events. We categorized eGFR and proteinuria into five (eGFR: ≥ 90, 60–89, 30–59, 15–29, and < 15 mL/min/1.73 m^2^) and four (UPCR: < 300, 300–999, 1000–2999, and ≥ 3000 mg/g), groups respectively. Then, we compared the risks of eMACEs and renal events among the 20 categories overall for eGFR and proteinuria, using the group with the greatest eGFR (≥ 90 mL/min/1.73 m^2^) and lowest proteinuria (< 300 mg/g) as the reference^2^. We assessed the independent associations of proteinuria as a continuous variable with the risks of eMACEs and renal events using Cox proportional hazard models. HRs were calculated for per 1-SD unit increase in UPCR. The multivariable Cox models were adjusted for age, sex, body mass index, smoking status, baseline eGFR, use of renin–angiotensin–aldosterone system blockers and statins, previous cardiovascular disease, and presence of diabetes, hypertension, or glomerulonephritis. The results are expressed as estimated hazard ratios (HRs) and 95% CIs. We examined the incidence of eMACEs according to log-transformed UPCR in an analysis stratified by urine creatinine concentration ≥ 95 or < 95 mg/dL by plotting an adjusted spline curve for the HR of the outcome. All statistical analyses were conducted using R software with various packages (version 3.5.3; The Comprehensive R Archive Network, http://cran.r-project.org).

### Patient and public involvement

Patients and/or the public were not involved in the design, or conduct, or reporting, or dissemination plans of this research.

## Supplementary Information

Supplementary Tables.

## Data Availability

Data are available on reasonable request. The corresponding author has full access to all data in the study and final responsibility for the submission of the article for publication. Due to data security reasons (ie, data contain potentially participant identifying information), the KNOW-CKD study does not allow sharing data as a public use file. Data requests can also be addressed to: jyjung@gachon.ac.kr.
