# Supplementary material for: Urine creatinine concentration influences the prognostic value of proteinuria for MACE prediction from the findings of the KNOW-CKD study
Source: Sci Rep. 2022 Sep 23;12:15924. doi: 10.1038/s41598-022-19819-9 (PMC9508084; doi:10.1038/s41598-022-19819-9)
Supplement: Supplementary file 1 — Supplementary Tables. [file 41598_2022_19819_MOESM1_ESM.pdf]

SUPPLEMENTARY MATERIALS

Title: Urine creatinine concentration influences the prognostic value of proteinuria for MACE prediction from the findings of the KNOW-CKD study

Authors' full names and affiliations:

Yun Jung Oh, MD, PhD[1,2]; Han Ro, MD, PhD[3,4]; Wookyung Chung, MD, PhD[3,4]; Young Youl Hyun, MD, PhD[5]; Sue Kyung Park, MD, PhD[6]; Yong-Soo Kim, MD, PhD[7]; Soo Wan Kim[8], MD, PhD; Yun Kyu Oh[9], MD, PhD; Kook-Hwan Oh, MD, PhD[10]; and Ji Yong Jung, MD, PhD[1,3,4]

[1]Department of Internal Medicine, Graduate School of Medicine, Gachon University, Incheon, Republic of Korea; [2]Division of Nephrology, Department of Internal Medicine, H Plus Yangji Hospital, Seoul, Republic of Korea; [3]Division of Nephrology, Department of Internal Medicine, Gachon University Gil Medical Center, Incheon, Republic of Korea; [4]Gachon University College of Medicine, Incheon, Republic of Korea; [5]Department of Internal Medicine, Sungkyunkwan University School of Medicine, Kangbuk Samsung Hospital, Seoul, Republic of Korea; [6]Department of Preventive Medicine, Seoul National University College of Medicine, Seoul, Republic of Korea; [7]Department of Internal Medicine, Seoul St. Mary's Hospital, Seoul, Republic of Korea; [8]Department of Internal Medicine, Chonnam National University Medical School, Gwangju, Republic of Korea; [9]Department of Internal Medicine, Seoul National University Boramae Hospital, Seoul, Republic of Korea; [10]Department of Internal Medicine, Seoul National University Hospital, Seoul, Republic of Korea

Correspondence to: **Ji Yong Jung, M.D., Ph.D.**

Mailing address: Division of Nephrology, Department of Internal Medicine, Gachon University Gil Medical Center, Gachon University College of Medicine, 21, Namdong-daero 774 beon-gil, Namdong-gu, Incheon, 21565, Republic of Korea

Tel: +82 32 458 2621

Fax: +82 32 460 3431

E-mail: jyjung@gachon.ac.kr

Supplemental Table 1. Adjusted hazard ratios of per SD-unit increase in urine albumin-creatinine ratio (UACR) for extended major adverse cardiovascular events (eMACEs) and renal events

**Overall (n=1,746)**		
	HR (95% CI)	*P*
eMACE	1.143 (0.979-1.334)	0.091
Renal event	1.809 (1.697-1.929)	<0.001
**eGFR ≥ 60 mL/min/1.73m^2 (n=618)**		
	HR (95% CI)	*P*
eMACE	2.109 (1.375-3.235)	0.001
Renal event	1.542 (1.170-2.032)	0.002
**eGFR :45-59 mL/min/1.73m^2 (n=284)**		
	HR (95% CI)	*P*
eMACE	1.495 (0.927-2.411)	0.099
Renal event	2.215 (1.758-2.791)	<0.001
**eGFR < 45 mL/min/1.73m^2 (n=844)**		
	HR (95% CI)	*P*
eMACE	1.074 (0.885-1.303)	0.469
Renal event	1.830 (1.701-1.968)	<0.001

Abbreviations: HR: hazards ratio; CI: confidence interval; UPCR, urine protein-creatinine ratio; eMACE, extended major adverse cardiovascular events.

*Adjusted for age, sex, BMI, smoking, previous CVD, diabetes, hypertension, gloemerulonephritis, baseline eGFR, and use of RAS blocker and statin.

Supplemental Table 2. Hazard ratios (with 95% confidence interval) for extended major adverse cardiovascular events (eMACEs) and renal events according to category of estimated glomerular filtration rate (eGFR) and urine albumin-creatinine ratio (UACR)

	Unadjusted HR (95% CI)				Adjusted HR* (95% CI)			
	UACR (mg/g)				UACR (mg/g)			
	<300	300-999	1000-2999	≥3000	<300	300-999	1000-2999	≥3000
**eMACE**								
≥90 mL/min/1.73m^2	Reference	1.33 (0.33-5.34)	1.25e-08 (0.00-inf)	7.92 (0.95-66.16)	Reference	1.50 (0.31-7.28)	2.47e-09 (0.00-inf)	2.16e+09 (0.00-inf)
60-89 mL/min/1.73m^2	2.59(1.00-6.67)	1.75(1.03-2.97)	1.23 (0.55-2.73)	3.40 (1.70-6.82)	1.50 (0.53-4.25)	1.49 (0.82-2.70)	1.65 (0.63-4.32)	5.87 (1.27-27.06)
30-59 mL/min/1.73m^2	1.67(1.07-2.60)	1.56 (1.15-2.11)	1.14 (0.78-1.66)	1.92 (1.35-2.74)	1.17 (0.69-1.98)	1.20 (0.81-1.76)	0.81 (4.82-1.35)	2.00 (0.99-4.04)
15-29 mL/min/1.73m^2	1.75 (1.27-2.40)	1.25 (0.96-1.63)	1.39 (1.08-1.79)	1.46 (1.09-1.97)	1.43 (0.94-2.16)	0.96 (0.69-1.33)	1.21 (0.84-1.74)	1.20 (0.79-1.85)
<15 mL/min/1.73m^2	1.14(0.67-1.94)	1.34 (1.04-1.73)	1.03 (0.00-inf)	1.32 (0.96-1.82)	1.01 (0.54-1.88)	1.08 (0.77-1.51)	0.02 (0.00-inf)	1.19 (0.72-1.97)
**Renal event**								
≥90 mL/min/1.73m^2	Reference	3.96 (1.92-13.18)	7.91 (2.06-30.31)	42.90 (4.14-149.80)	Reference	10.11 (1.47-69.68)	4.44 (0.76-26.04)	4.31e+09 (4.44e+08-4.19e+10)
60-89 mL/min/1.73m^2	4.26 (1.58-11.50)	3.05 (1.69-5.50)	4.87 (2.74-8.65)	5.69 (2.69-12.03)	6.81 (2.17-21.44)	3.86 (1.78-8.39)	6.51 (3.25-13.02)	13.24 (6.07-28.91)
30-59 mL/min/1.73m^2	2.86 (1.80-4.55)	2.75 (2.02-3.74)	3.40 (2.50-4.61)	4.50 (3.02-6.73)	4.36 (2.63-7.22)	3.08 (2.15-4.41)	3.59 (2.57-5.02)	4.76 (3.16-7.17)
15-29 mL/min/1.73m^2	2.90 (2.12-3.98)	2.81 (2.22-3.54)	3.56 (2.65-4.78)	3.29 (2.56-4.22)	3.04 (2.13-4.34)	2.92 (2.26-3.79)	3.64 (2.64-5.02)	4.23 (2.78-6.44)
<15 mL/min/1.73m^2	4.17 (2.50-6.95)	3.12 (2.32-4.19)	2.97 (2.31-3.82)	3.53 (2.3305.33)	4.71 (2.66-8.35)	4.25 (3.06-5.91)	2.96 (2.28-3.82)	11.40 (3.63-35.79)

Abbreviations: HR: hazards ratio; CI: confidence interval; UPCR, urine protein-creatinine ratio; eMACE, extended major adverse cardiovascular events.
*Adjusted for age, sex, BMI, smoking, previous CVD, diabetes, hypertension, gloemerulonephritis, and use of RAS blocker and statin.

Supplemental Table 3. Adjusted hazard ratios of per SD-unit increase in urine albumin-creatinine ratio (UACR) for extended major adverse cardiovascular events (eMACEs) and renal events

**Overall (n=1,746)**			
	HR (95% CI)	*P*	
eMACE	1.134 (0.972-1.323)	0.111	
Renal event	1.761 (1.656-1.873)	<0.001	
**eMACE**			
		P	
	HR (95% CI)	effect	interaction
eGFR ≥60 mL/min/1.73m^2 (n=618)	2.107 (1.388-3.197)	0.001	0.010
eGFR <60 mL/min/1.73m^2 (n=1,128)	1.069 (0.895-1.277)	0.461	
**Renal event**			
		P	
	HR (95% CI)	effect	interaction
eGFR ≥60 mL/min/1.73m^2 (n=618)	1.600 (1.224-2.092)	0.001	0.086
eGFR <60 mL/min/1.73m^2 (n=1,128)	1.790 (1.677-1.910)	<0.001	

Abbreviations: HR: hazards ratio; CI: confidence interval; UPCR, urine protein-creatinine ratio; eMACE, extended major adverse cardiovascular events.

*Adjusted for age, sex, BMI, smoking, previous CVD, diabetes, hypertension, gloemerulonephritis, baseline eGFR, and use of RAS blocker and statin.

Supplemental Table 4. Adjusted hazard ratios of per SD-unit increase in urine albumin-creatinine ratio (UACR) for extended major adverse cardiovascular events (eMACEs) and renal events stratified by urine creatinine concentration across the whole study population

**eGFR ≥60 mL/min/1.73m^2 (n=618)**							
eMACE				Renal event			
		P				*P*	
	HR (95% CI)	effect	interaction		HR (95% CI)	effect	interaction
Urine creatinine ≥ 95mg/dL	2.366 (1.348-4.154)	0.003	0.983	Urine creatinine ≥ 95mg/dL	1.697-1.183-2.433)	0.004	0.776
Urine creatinine < 95mg/dL	2.582 (1.156-5.767)	0.021		Urine creatinine < 95mg/dL	2.061 (1.208-3.517)	0.008	
**eGFR <60 mL/min/1.73m^2 (n=1,128)**							
eMACE				Renal event			
		P				*P*	
	HR (95% CI)	effect	interaction		HR (95% CI)	effect	interaction
Urine creatinine ≥ 95mg/dL	1.327 (0.956-1.841)	0.091	0.082	Urine creatinine ≥ 95mg/dL	1.858 (1.644-2.101)	<0.001	<0.001
Urine creatinine < 95mg/dL	1.015 (0.818-1.259)	0.894		Urine creatinine < 95mg/dL	1.772 (1.635-1.920)	<0.001	

Abbreviations: HR: hazards ratio; CI: confidence interval; UPCR, urine protein-creatinine ratio; eMACE, extended major adverse cardiovascular events.
*Adjusted for age, sex, BMI, smoking, previous CVD, diabetes, hypertension, gloemerulonephritis, baseline eGFR, and use of RAS blocker and statin.
